# Enhancing Caregiver Empowerment Through the Story Mosaic System: Human-Centered Design Approach for Visualizing Older Adult Life Stories

**DOI:** 10.2196/50037

**Published:** 2023-11-08

**Authors:** Fang Gui, Jiaoyun Yang, Qilin Wu, Yang Liu, Jia Zhou, Ning An

**Affiliations:** 1 Key Laboratory of Knowledge Engineering with Big Data, Ministry of Education, Hefei, China; 2 School of Computer Science and Information Engineering, Hefei University of Technology, Hefei, China; 3 School of Computer Science and Artificial Intelligence, Chaohu University, Hefei, China; 4 Department of Industrial Engineering, Chongqing University, Chongqing, China

**Keywords:** life story visualization, Story Mosaic system, human-centered design, prototype refinement workshops, eldercare, caregiver, elder, older adult

## Abstract

**Background:**

Various older adult care settings have embraced the use of the life story approach to enhance the development of comprehensive care plans. However, organizing life stories and extracting useful information is labor-intensive, primarily due to the repetitive, fragmented, and redundant nature of life stories gathered from everyday communication scenarios. Existing life story systems, while available, do not adequately fulfill the requirements of users, especially in the application of care services.

**Objective:**

The objective of this study is to design, develop, and evaluate a digital system that provides caregivers with the necessary tools to view and manage the life stories of older adults, enabling expedited access to pertinent information effectively and visually.

**Methods:**

This study used a multidisciplinary, user-centered design approach across 4 phases: initial design requirements, prototyping, prototype refinement workshops, and usability testing. During the initial phase, we conducted field research in the Hefei Tianyu Senior Living Service Nursing Home, China, to discover how caregivers currently store and use life stories and their needs, challenges, and obstacles in organizing and retrieving information. Subsequently, we designed a low-fidelity prototype according to the users’ requirements. A prototyping workshop involving 6 participants was held to collaboratively design and discuss the prototype’s function and interaction. User feedback from the workshops was used to optimize the prototype, leading to the development of the system. We then designed 2 rounds of usability testing with 7 caregivers to evaluate the system’s usability and effectiveness.

**Results:**

We identified 3 categories of functionalities that are necessary to include in the design of our initial low-fidelity prototype of life story visualizations: life story input, life story organization, and timeline generation. Subsequently, through the workshops, we identified 3 categories for functional optimization: feedback on user interface and usability, optimization suggestions for existing features, and the request for additional functionalities. Next, we designed a medium-fidelity prototype based on human-centered design. The Story Mosaic system underwent usability testing in the Hefei Tianyu Senior Living Service Nursing Home. Overall, 7 users recorded and organized 1123 life stories of 16 older adults. The usability testing results indicated that the system was accessible and easy to use for caregivers. Based on the feedback from the usability testing, we finalized the high-fidelity prototype.

**Conclusions:**

We designed, developed, and evaluated the Story Mosaic system to support the visual management of older adults’ life stories. This system empowers caregivers through digital technology and innovative design, pioneering personal narrative integration in caregiving. This system can expand to include informal caregivers and family members for continued adaptability and empathy.

## Introduction

### Background

The growing aging population is projected to reach 1.5 billion by 2050, with around 50% of older adults experiencing loneliness and social isolation, which poses serious public health risks and is associated with adverse health outcomes [1]. The use of life stories as a medium for communication with older adults has evolved into a potent intervention that is capable of mitigating their social obstacles and enhancing their quality of life [2]. Professional caregivers can benefit from this approach by engaging older adults to talk about their life experiences, extracting meaningful messages from these stories, and applying these insights to personalized care for these older adults [3].

Some nursing homes have started incorporating older individuals’ life stories into their health information archives [4]. During the admission process, through communication with older adults or their family members, information is gathered about their life experiences, interests, hobbies, social relationships, and more [5]. These data serve as the foundation for personalized care plans, aligning with the person-centered care approach [6]. However, life stories in daily settings are rarely narrated in their entirety but are often fragmented and disorganized [7], making interpretation and application by caregivers challenging.

Although most of the existing research has relied on the manual organization of the life stories of older adults [8], there is an effort to automate this process [9]. Even when automated, the lack of effective visualization hinders the full realization of life stories’ potential, marking a gap in using life stories in caregiving practice.

### Objective

This study sought to design, develop, and evaluate the Story Mosaic system, a tool specifically crafted to visually represent the life stories of older adults for professional caregivers. The Story Mosaic system intends to facilitate efficient information extraction, enable automated organization, and promote enhanced management of these narratives. By rationally organizing life stories, the presentation becomes more straightforward and intuitive, aiding caregivers in better understanding older adults, having an insight into the needs of older adults, and providing personalized care. The system enables life stories to continue to enrich and grow dynamically as caregivers interact more deeply with older adults, fostering a virtuous service cycle.

**Figure 1.**
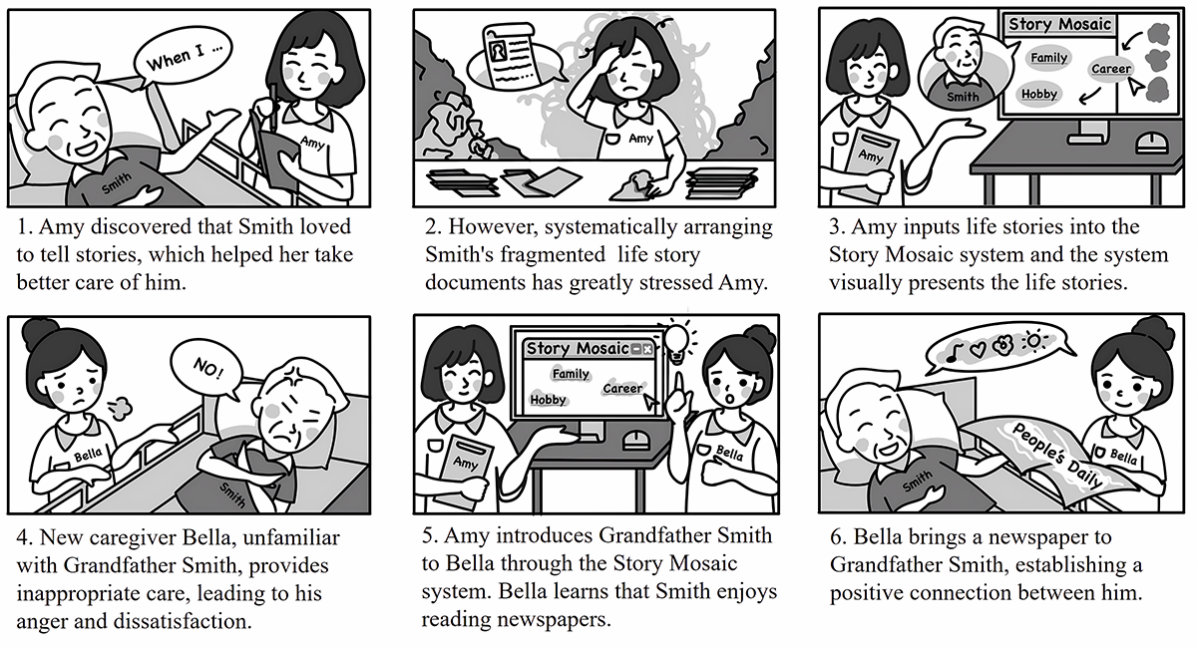
A big-picture storyboard for the Story Mosaic system.

We drew a use case, as shown in Figure 1, to describe the Story Mosaic system’s users and features and the real-world problem it solves. The system is designed for professional caregivers involved in the daily care of older adults. The life stories that older adults share with their caregivers are often fragmented and marked by redundancy and disarray, creating considerable challenges in their organization and interpretation. Caregivers can input older adults’ life stories into the Story Mosaic system in batches. The system performs tasks such as categorizing stories by themes, arranging them chronologically, and generating a timeline. It visualizes crucial information within these life stories, providing caregivers with data to offer personalized services to older adults.

## Methods

### Ethical Considerations

Approval was obtained from the research ethics board of the Hefei University of Technology (HFUT20220921001). All participants provided informed consent and participated voluntarily; no compensation was involved. The information and pictures of older adults included in this study adhere to privacy policies.

### Study Overview

**Figure 2.**
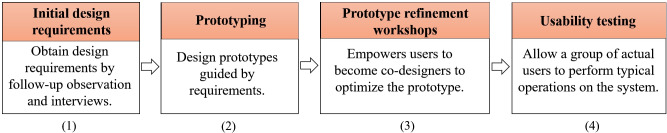
The implementation of the Story Mosaic system: (1) initial design requirements, (2) prototyping, (3) prototype refinement workshops, and (4) usability testing.

This study used human-centered design (HCD) methodology to ensure the efficacy and efficiency of the final product for the intended user population [10]. In this study, we implemented the Story Mosaic system using a 4-phase strategy consisting of initial design requirements, prototyping, prototype refinement workshops, and usability testing, as shown in Figure 2.

### Software Language

When analyzing interview and observational data, we used the speech-to-text tool provided by iFlytek (China Mobile) [11] and Quirkos (Quirkos Software) [12] to expedite data coding and analysis. For the prototype design, we used the collaborative web-based prototyping tool, MoDao (Wondershare) [13], to facilitate the design process among multiple team members. In developing the system, we used Node.js (OpenJS Foundation) [14] and TypeScript (Microsoft) [15] to construct the web server, providing a robust platform for handling incoming requests.

### Phase 1: Initial Design Requirements

We conducted field research between February and April 2023 in the Hefei Tianyu Senior Living Service Nursing Home, China. The aim was to comprehensively understand the initial design requirements that should be included in the Story Mosaic system.

#### Recruitment

We recruited 2 administrators and 3 caregivers. The inclusion criteria for the participants were (1) more than 2 years of nursing experience and (2) current participation in life story intervention in older adult care. Table 1 displays the characteristics of the participants in acquiring the initial design requirements.

**Table 1.** Participants’ characteristics in initial design requirements acquisition.

ID	Identity	Sex	Age (years)	Years of older adult care
A1	Administrator	Male	34	10
A2	Administrator	Female	26	3
C1	Caregiver	Female	53	7
C2	Caregiver	Female	42	6
C3	Caregiver	Male	33	2

#### Procedure

We conducted a follow-up observation, supplemented by 5 semistructured one-on-one interviews during the observation [16,17]. The follow-up observation was to observe the 3 caregivers interacting with older adults in clinical care and the way they organized and looked up their life stories. The 5 semistructured one-on-one interviews were conducted with administrators and caregivers to gain insight into their difficulties in organizing the life stories of older adults and their design needs for a life story visualization system. Based on the data analysis results of the follow-up observation, we mainly discussed the following questions with the participants in the semistructured interview:

1. What types of life stories do older adults usually tell in daily interactions?
2. How do you generally organize the life stories of older adults? What are the difficulties?
3. When you look it up again, can you quickly find the target life story?
4. What do you hope the Story Mosaic system can provide you?
5. What do you think is the most essential function of the Story Mosaic system?

#### Data Analysis

The above tasks were audio recorded with participant consent and transcribed into words by team members to familiarize themselves. The team members then annotated and categorized the data based on the observations, initially identifying distinct user requirement themes [18]. Subsequently, data from 3 semistructured interviews were chosen for in-depth analysis to validate whether the identified themes aligned with the findings from the follow-up observation. Necessary adjustments and enrichments were made to the themes during this process. Subsequently, the theme framework was further validated using data from 2 additional semistructured interviews.

### Phase 2: Prototyping

#### Low-Fidelity Prototype

**Figure 3.**
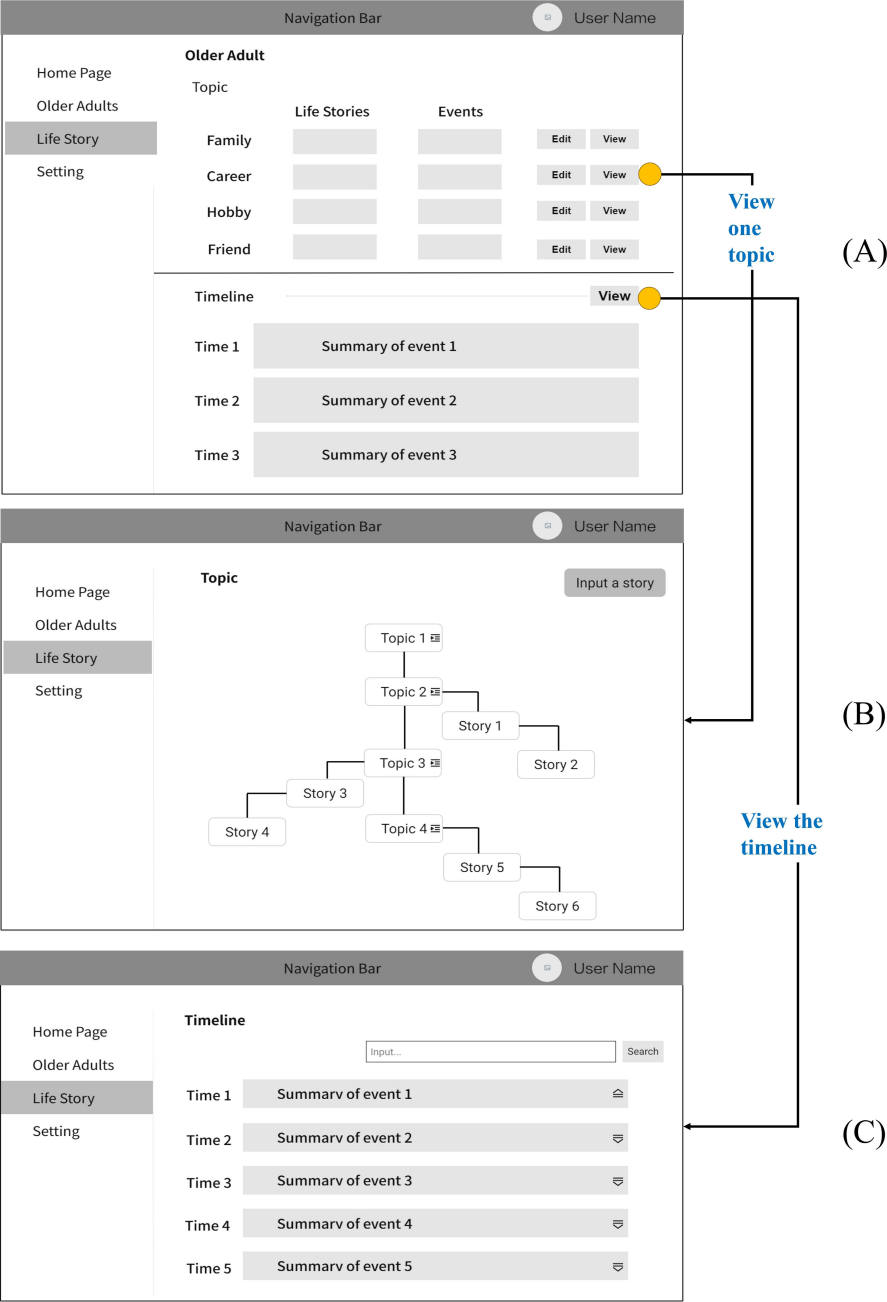
Low-fidelity prototype mock-ups of the Story Mosaic system. (A) The visual interface for older adults’ life stories is bifurcated into 2 distinct modules: topic and timeline. (B) Detailed diagram of the topic module in (A), where life stories are organized hierarchically by topics. The topics form the trunk, and the life stories are the branches beneath each topic. (C) Detailed diagram of the timeline module in (A), displaying the older adult’s important life experiences as a timeline, with each node representing a key event from their life story.

We designed a low-fidelity prototype (Figure 3) of the Story Mosaic system with the initial design requirements. The mapping relationship between initial design requirements and prototype functions is shown in Multimedia Appendix 1. The system completes tasks including organizing stories chronologically, categorizing them according to topics, and creating a timeline. It depicts crucial information from these life stories, giving caregivers information they can use to provide individualized care for older adults.

#### Procedure

We discussed and iterated sketches of the system within our multidisciplinary team, which included 2 user experience designers, 2 computer science researchers, and 2 caregivers (C1 and C2, who participated in the initial design requirements study). Once we finalized the initial design of our system, we converted the sketches to a digital low-fidelity prototype using MoDao (a prototyping tool that facilitates collaborative web-based interaction among multiple users).

Given the relatively older age of caregivers [19], we adopted an age-friendly interaction design based on HCD during prototyping [20-22].

Regarding font size and colors, our design used large font sizes and warm colors to foster a friendly interface [23].

We considered features to make the system easy to click. As individuals age, they may experience a decline in hand-eye coordination and motor functions, making it challenging for them to interact with a user interface (UI) [24]. Tasks such as clicking on interface targets, moving between interface elements, and responding to on-screen targets become difficult. We addressed this difficulty by ensuring that the interactive UI components were sufficiently large with a minimum diagonal length of 11 mm and adequately distanced from other elements with at least a 2-mm gap. Additionally, scroll bars could pose usability issues. The system was designed to simplify scroll bars and provide users with alternative options such as clicking on scroll bar arrows, using the keyboard’s up and down keys, and having action buttons that return them directly to the top of the page [25].

Lastly, we aimed to have consistent layouts. We established a well-defined grid system during the prototype design phase to maintain consistent spacing and alignment. We adhered to a unified typography and color scheme while consistently implementing a standardized set of UI elements and components across the entirety of the design [26].

### Phase 3: Prototype Refinement Workshops

In this phase, we organized participatory design workshops to refine system prototypes. Participatory design is a method that empowers users to become co-designers, actively involving them throughout the design process [27].

#### Recruitment

We recruited 6 participants for the workshops on the Story Mosaic system prototype, comprising 1 UI designer, 1 aging-product designer, and 4 caregivers. In total, 2 of the 4 caregivers were participants in the phase-1 study, and the other 2 were new participants. Table 2 displays the characteristics of the participants in the prototype refinement workshops.

**Table 2.** Participants’ characteristics in the prototype refinement workshops.

ID	Identity	Sex	Age (years)	Years of older adult care
D1	UI^a^ designer	Male	33	N/A^b^
D2	Aging-product designer	Female	42	6
C1	Caregiver	Female	53	7
C2	Caregiver	Female	42	6
C4	Caregiver	Male	32	3
C5	Caregiver	Female	28	3

a UI: user interface.

b N/A: not applicable.

#### Procedure

The primary objective of these design workshops was twofold: to acquire feedback on the prototype and to identify unanticipated requirements. Before commencing the participatory design workshops, we obtained informed consent and demographic information from the participants. We conducted 4 workshops with the same group of participants, each consisting of 6 participants, to acquire feedback on the content, presentation, and interaction of our proposed prototype. We conducted participatory workshops with the same group of participants every 2 weeks at the nursing home, recording the audio of each session. The initial 3 workshops focused on seeking feedback and suggestions on different parts of the prototype [28]. After analyzing the data gathered in the first 3 design workshops, we designed an interactive medium-fidelity prototype, ensuring that all issues raised during the initial 3 workshops were addressed. Finally, we presented the medium-fidelity prototype in the fourth workshop, followed by a final round of feedback.

#### Data Analysis

We conducted a thematic analysis of the prototype refinement workshops. The primary objective of this analytical process was to synthesize the feedback received concerning our prototype and to unveil latent user requisites that may have been overlooked during the initial phase of the study. We transcribed the proceedings of the initial 3 design workshops, leading to the inception of preliminary themes. Subsequently, we used the records from the fourth design workshop for validation and refinement. Finally, we curated illustrative quotations corresponding to each thematic category and succinctly encapsulated our discoveries. We refrained from presenting quantitative details, such as enumerating the frequency of information occurrences. The objective of this phase was to unveil unexpected insights through an inductive approach to data collection and analysis [29].

### Phase 4: Usability Testing

It is imperative to consider whether usability testing would produce anything meaningful in the system design stage [30].

#### Recruitment

We recruited 7 participants to participate in usability testing. Table 3 displays the characteristics of the participants in the usability tests.

**Table 3.** Participants’ characteristics in the usability tests.

ID	Identity	Sex	Age (years)	Years of older adult care
A1	Administrator	Male	34	10
A2	Administrator	Female	26	3
C3	Caregiver	Male	33	2
C6	Caregiver	Female	45	3
C7	Caregiver	Male	23	1
C8	Caregiver	Female	54	5
C9	Caregiver	Female	45	3

#### Procedure

We conducted observational studies to better understand users’ natural interactions with the product, monitoring users as they engaged with the Story Mosaic system. Following literature guidelines [31], we adopted an iterative approach for our usability tests, dividing them into batches. The initial test involved 3 participants, and we addressed their predominant usability issues. We then tested the system again with 4 participants. The moderator asked the participants to use different functionalities within the system while reflecting on the experience of using life stories. During the usability tests, the moderator noted any difficulties the participants encountered. The participants filled out the System Usability Scale (SUS) at the end of the usability tests. We deployed the Story Mosaic system in the Hefei Tianyu Senior Living Service Nursing Home for practical use verification. The usability task and interview questions are presented in Multimedia Appendix 2.

#### Data Analysis

We systematically analyzed and categorized user interactions with our system during each task. The *Results* section details the usability test results and provides the associated optimization recommendations.

## Results

### Phase 1: Initial Design Requirements

We preliminarily identified 3 categories of functionalities within the life story visualization module of the system: (1) life story input, (2) life story organization, and (3) timeline generation. Caregivers can input multimodal life stories (text, picture, video, etc) into the system, and during the input process, they can annotate elements such as time. Subsequently, the system categorizes and organizes life stories by themes, producing a thematic visual timeline for older adults. We established the initial design requirements list, as shown in Multimedia Appendix 3.

### Phase 2: Prototyping

#### Overview

The prototype of the Story Mosaic system primarily encompasses functionalities within 3 modules: older adults management, life story management, and system settings. The life story management module is the core of the Story Mosaic system, and we describe it in detail in the following section. The life story management module contains 3 important functions: life story input, automated life story organization, and timeline generation. The mapping relationship between initial design requirements and prototype functions is shown in Multimedia Appendix 1. The low-fidelity prototype of the life story management module is shown in Figure 3.

#### Life Story Input

The life story input module empowers users to manually input pivotal components, including time, location, individuals, and event summaries, with the flexibility of these particulars being nonmandatory. Moreover, the platform provides an algorithmic mechanism for automated padding in instances where the above information is absent. Users can enrich life stories with various formats, encompassing textual content, images, and videos. Furthermore, the system supports theme personalization, enabling users to establish, modify, eliminate, and retrieve the topics for older adults’ life stories within the framework.

#### Automated Life Story Organization

In the low-fidelity prototype, we established 5 primary themes: “Friends and Family,” “Career,” “Achievement,” “Marriage,” and “Interests.” It is within the prerogative of users to exercise adaptability, enabling the customization and introduction of novel themes, all contingent on the idiosyncratic life experiences of older adults. The automated life story organization module allows users to classify life stories through manual themes specification. In the absence of specified themes, the system defaults to an algorithm for automated organization. Narratives are methodically sequenced based on temporal attributes, from earlier experiences to the most recent events. Additionally, the life story presentation interface incorporates a search box, facilitating the execution of fuzzy searches across these narratives.

#### Timeline Generation

**Figure 4.**
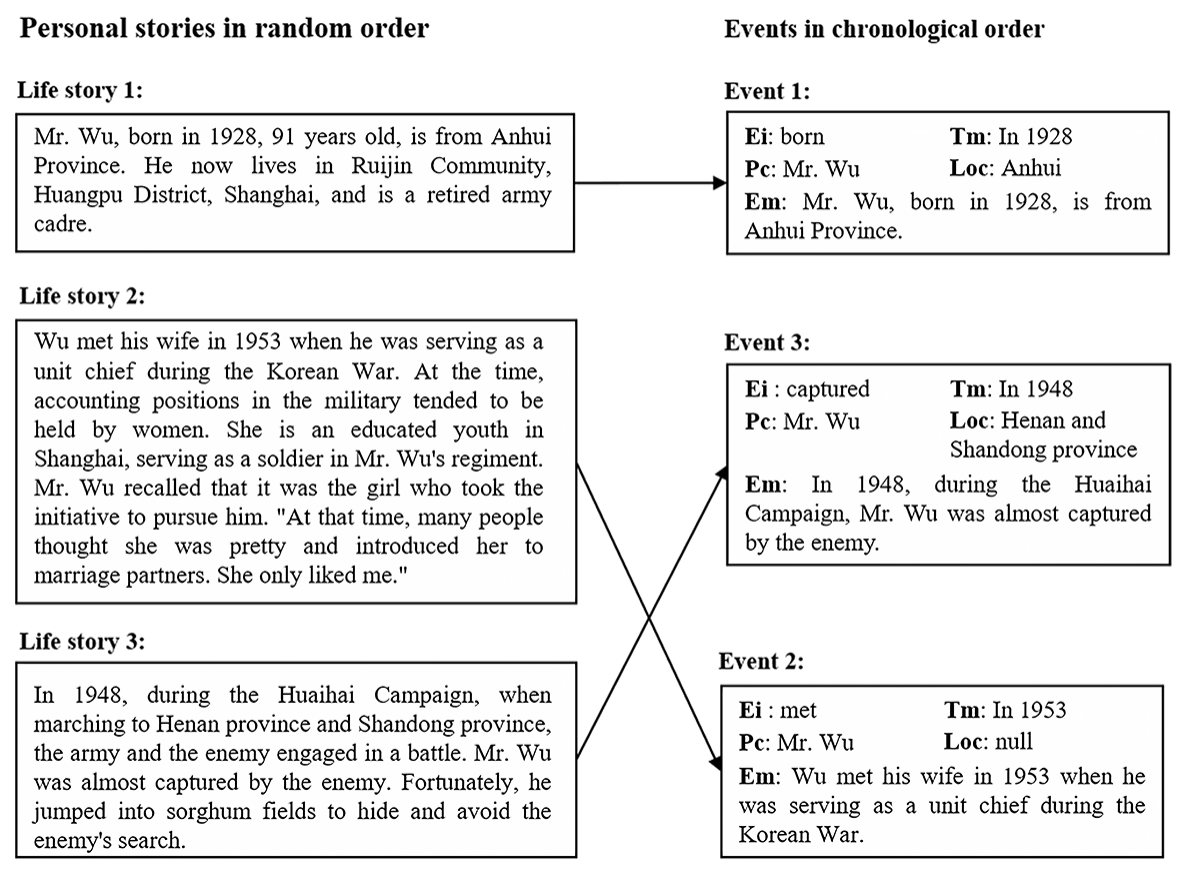
The timeline generation process extracts the crucial event from each life story and sorts them chronologically. Ei: event induction; Em: event mention; Loc: location; Pc: participant; Tm: time.

The primary objective of the timeline generation module is to construct a chronological framework of older adults’ life journey, furnishing caregivers with a swift avenue to comprehend the older adult. The core functionality of this module revolves around streamlining details within the older adult’s life narratives, highlighting pivotal events within each life story. Subsequently, the crucial events are organized based on chronological order, generating a cohesive timeline. Each node on the timeline is represented as “Time: Story Summary.” Figure 4 shows an example of the timeline generation process [32].

### Phase 3: Prototype Refinement Workshops

#### Overview

**Figure 5.**
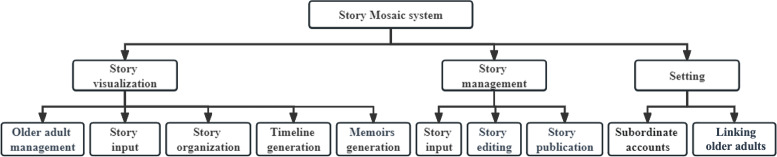
The functional architecture diagram of the Story Mosaic system.

**Figure 6.**
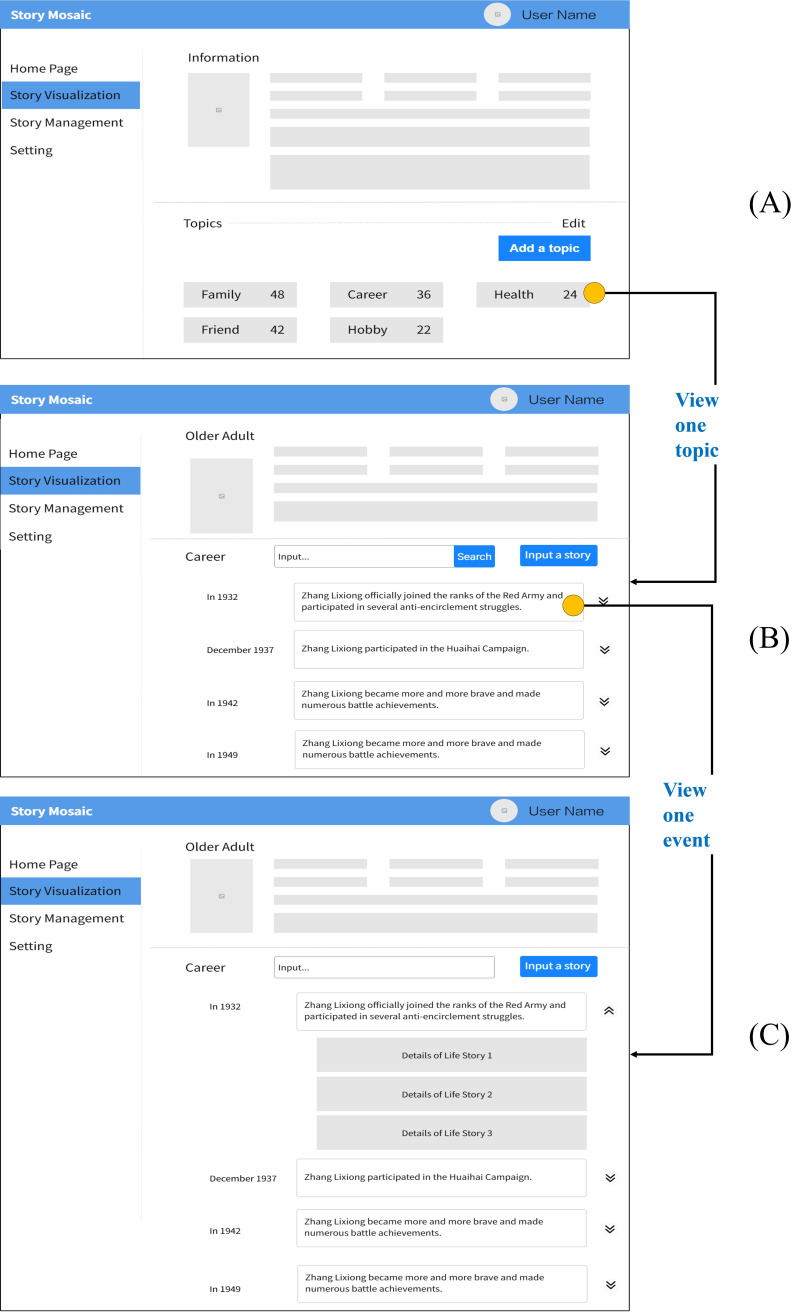
Medium-fidelity prototype mock-ups of the Story Mosaic system. (A) The interface visually depicts the older adult’s life story. Using workshop feedback, the top section displays vital information, easing caregiver memory strain. The lower part emphasizes life story themes and quantities while integrating content from the timeline module into the topic module. (B) By clicking on a specific theme, users can access the viewing interface for life stories associated with that theme. The visual representation of life stories from the low-fidelity prototype is harmoniously integrated with the timeline display, resulting in a reduction of user interactions. (C) Caregivers can click on a specific event node within the timeline to view all fragmented life stories associated with that event node.

The participants had valuable insights and feedback on the prototype, which helped us to identify several areas for functional design and improvement during the prototype refinement workshops. We also discovered some unanticipated requirements. We analyzed the results of the prototype refinement workshops to establish the functional architecture of the Story Mosaic system and design the medium-fidelity prototype. The functional architecture diagram is shown in Figure 5. The medium-fidelity prototype of the story visualization, which is the most important module of the Story Mosaic system, is shown in Figure 6. Multimedia Appendix 4 provides the feedback analysis of the workshops.

#### Visualization of Story Mosaic

Visualizing older adults’ life stories is a central aspect of the Story Mosaic system. In the prototype refinement workshops, we extensively discussed the visualization methods with the participants. Considering their work requirements and suggestions, we iteratively optimized the forms of visualizing life stories. Table 4 presents the participants’ perspectives on the strengths, weaknesses, and improvement suggestions for each visualization approach in the prototype refinement workshops.

**Table 4.** Participants’ comments and suggestions of the story mosaic.

Approach	Advantages	Disadvantages	Improvement suggestions
A	The hierarchical structure, consisting of a main trunk and branches, can effectively assist caregivers in organizing the life trajectory of older adults [C1 and D2]	It hinders the expansion of life stories and results in crowded content when displaying story details within the nodes [D2]	Maintain the hierarchical structure while adopting a zigzag-shaped multilevel visualization approach [D1 and D2]
B	Organize life stories according to chronological or logical rules, maintaining a clear hierarchical structure that facilitates the web-based expansion of life stories [D1, C3, and C4]	The presence of potential information overload The absence of prominent highlighting of essential information, resulting in significant reading barriers [D1 and C3]	Implement filters and sorting options to manage and retrieve data effectively Differentiate important information by using various colors based on the frequency of story occurrences [D2 and C4]
C	Retain approach B’s advantages while highlighting important information with colors Comprehensive and detailed [C2 and C5]	Lack of interactivity Complex content may confuse users [D1 and C4]	Incorporate interactive elements to enhance user engagement and exploratory behavior Simplify the interface without sacrificing important information [C3 and D2]
D	Efficient and well organized Visually appealing User-friendly with simple and intuitive interactions [C1, C4, and C5]	The customization options are limited in scope [C2 and C5]	The participants expressed satisfaction with this approach and did not suggest improvements

#### Feedback on UI and Usability

Participants offered insights regarding the system’s usability and visual design. They indicated a desire for clear instructional cues, such as an evident “click here to return” prompt during page navigation. They also noted difficulties in interacting with the system’s button, primarily when solely represented by icons or text. A preference emerged for buttons combining icons and text, accompanied by an adequately sized font. Moreover, the frequency of retelling life stories carries varying implications for an individual. Stories mentioned often indicate events that had a profound impact or left a strong impression on the older adult, thus forming suitable topics to initiate engagement in care. On the other hand, seldom recounted experiences could signify events the individual prefers not to discuss, suggesting possible areas necessitating tactful handling in their care plan. Responding to feedback from nursing home caregivers who participated in the survey, we adopted the progressive color representation method.

#### Request for Additional Functionalities

The following additional functionalities were requested. (1) The life story management function was deemed valuable, designed to preserve life stories that have yet to be included in the life story structure. Users can add, delete, and review life stories in the story bank. (2) The memoir function was desired to enable users to choose some life stories from the life story structure and create an electronic memoir for older adults or their family members. (3) An interface for older adults’ children was also deemed beneficial. The system management module of older adults’ care homes could incorporate individual accounts for older adults’ children. This could let them see caregiver efforts for their parents and help strengthen parent-child bonds through exploring life stories.

### Phase 4: Usability Testing

#### Overview

**Figure 7.**
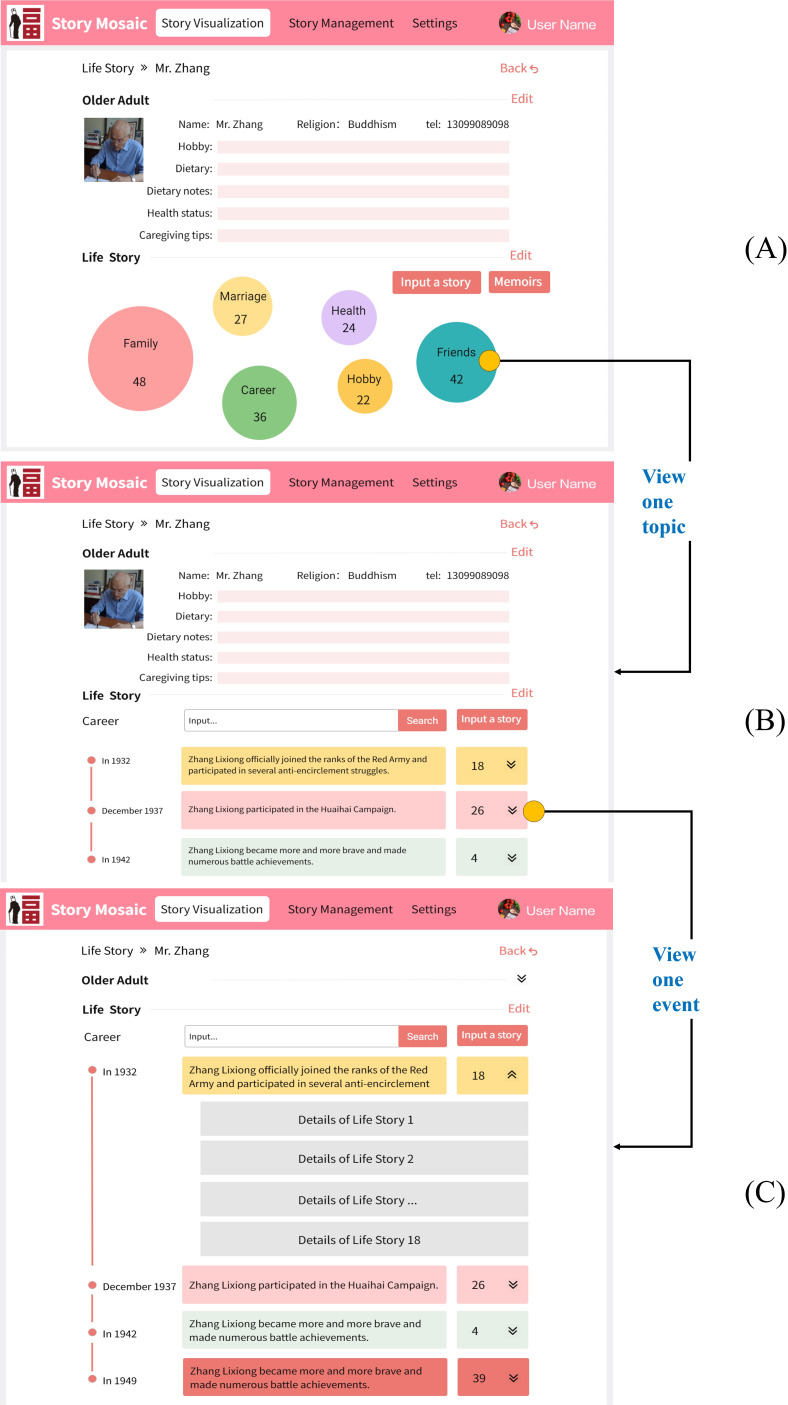
High-fidelity prototype mock-ups of the Story Mosaic system. (A) The interface visually presents the life story of the older adult. Different themes are represented by different colored circles, and the size of the circle is proportional to the number of life stories it contains. (B) By clicking on a specific theme, users can access the viewing interface for life stories associated with that theme. Different event nodes have different color backgrounds. As the life stories contained under an event become more fragmented, the background color of the event node becomes darker. (C) Caregiver can click on a specific event node within the timeline to view all fragmented life stories associated with that event node.

The Story Mosaic system underwent usability testing in the Hefei Tianyu Senior Living Service Nursing Home. In all, 5 caregivers and 2 administrators collaborated on inputting 1123 life stories from 16 older adults into the system. The usability task and interview questions are presented in Multimedia Appendix 2. In the usability testing feedback, we summarized the following issues: interaction, bootstrap operation, input errors, and complex operation. Multimedia Appendix 5 details the problems encountered by participants during usability testing and our proposed fixes. We analyzed the results of usability testing to design the high-fidelity prototype. Figure 7 is the high-fidelity prototype of the life story organization and visualization, and the home page of the Story Mosaic system is shown in Multimedia Appendix 6.

#### Interaction Operation

Some participants raised concerns about whether the interactive functions were identifiable. For example, the search button represented by a magnifying glass icon was not recognized, or instead of clicking the “view” button, they clicked directly on the story to view its details. It was also difficult for some to view the target life stories. To address these issues with interactive operation, we made the following optimizations:

1. For icons representing “search,” “add,” and other operations, we used a combination of icons and text.
2. We increased the interaction area for viewing life stories so that users could click anywhere on the life story to view its details.
3. The system supported a search function for life stories, allowing users to search for target events based on keywords.

#### Bootstrap Operation

By observing the participants’ actions, we identified opportunities for improvement in the guided processes. For example, participants exhibited prolonged hesitation when returning to the previous page from the current interface, and they overlooked the “one-click return to top” button on the page when scrolling to the bottom while viewing life stories. In response to the usability issues encountered in the guided operations, we implemented the following optimizations:

1. Besides the system menu navigation, we added breadcrumb navigation to each interface in the navigation settings.
2. When users scroll to the middle or bottom of the story-viewing interface, the “one-click return to top” button would be highlighted, prompting users to quickly return to the top of the page from this location.

#### Input Errors

Some participants made input errors when adding their life stories, such as ignoring the theme selection. Therefore, in all input operations of the system, input prompts, input warnings, and input error prompts were added. We marked the operation prompt as “required*” for all required fields, to indicate that they must be filled in.

#### Complex Operation

To prevent batch upload failures, the system offered format guidelines, sample files, and preupload format verification with informative error messages for any related issues. Furthermore, we improved password handling by eliminating the need for both uppercase letters and symbols, and we provided accessible guidance for password recovery and resetting.

In the study follow-up, participants completed the SUS, an industry-standard tool offering a holistic measure of usability [33]. As shown in Table 5, the first set of SUS scores was 70.00, 75.00, and 75.00 (mean 73.33), achieving a B– on the SUS grade and indicating higher usability than 65% of other websites. The second set of SUS scores was 82.50, 77.50, 80.00, and 82.50 (mean 80.63), achieving an A– on the SUS grade and indicating higher usability than 85% of other websites. These responses indicate that participants felt the tool was intuitive and valuable enough to use frequently.

**Table 5.** System Usability Scale (SUS) scores from 2 usability evaluations

Item	First usability evaluation, SUS score	Second usability evaluation, SUS score
1. I think that I would like to use this system frequently.	3.33	3.75
2. I found the system unnecessarily complex.	1.67	1.50
3. I thought the system was easy to use.	3.67	4.00
4. I think that I would need the support of a technical person to be able to use this system.	1.67	1.50
5. I found the various functions in this system were well integrated.	3.67	3.75
6. I thought there was too much inconsistency in this system.	1.67	1.50
7. I would imagine that most people would learn to use this system very quickly.	3.67	4.25
8. I found the system very cumbersome to use.	1.67	1.50
9. I felt very confident using the system.	4.00	4.00
10. I needed to learn a lot of things before I could get going with this system.	2.33	1.50

## Discussion

### Principal Findings

This study presents the design, development, and evaluation of the Story Mosaic system’s prototypes, aiming to empower caregivers to harness valuable insights from older adults’ life stories through a structured 4-phase HCD approach.

In phase 1 (initial design requirements), evidence revealed a substantial gap in digital tools that can organize and visually depict life narratives, despite the growing use of life stories in older adult care. This deficit could inadvertently intensify caregiver workloads, underscoring the urgent need for practical digital solutions.

In phase 2 (prototyping) and phase 3 (prototype refinement workshops), we progressed from low-fidelity and medium-fidelity to high-fidelity prototypes. The Story Mosaic system has evolved to include modules for life story management, life story visualization, memoir generation, and system settings. Our team’s diversity, consisting of professional caregivers and user experience designers, was instrumental in refining a practical, user-centric system. Feedback in phase 3 accentuated caregivers’ design preferences, highlighting larger fonts, clear color contrasts, ample-sized buttons, and uniform layouts. Participants were keenly engaged in discussing the visual organization of life stories. They offered essential suggestions for optimizing the visual organization of life stories, emphasizing hierarchy, simplification, and minimized complexity.

In phase 4 (usability testing), the results validated the system’s efficacy in addressing challenges in manually organizing and retrieving life stories, marking it as a pertinent instrument for personalized care.

Although the feedback trended positively, we recognized specific design shortcomings, including interaction operation, bootstrap operation, input errors, and complex operation. Moving forward, it is imperative to address these challenges, particularly when crafting digital tools for the older caregiver demographic.

### Limitations

Our study has noteworthy limitations. Primarily, although the Story Mosaic system is designed to organize and extract pertinent details from life stories in a text format, we discovered that audio recordings tend to be the preferred mode for capturing these narratives. Thus, a focal point of our subsequent work will involve efficient collection methods for life stories. We envision the future integration of the Story Mosaic system with digital mobile storytelling tools, mitigating the challenges caregivers currently encounter when manually inputting life narratives.

Furthermore, the application of emerging natural language processing techniques for organizing and extracting information from life story text remains in its infancy. Such techniques may occasionally generate unintended outcomes [34], which can inadvertently influence caregivers’ perceptions of older adults. However, our confidence in the system’s potential is bolstered by its application in the Tianyu Senior Living Service Nursing Home. We remain committed to refining the system based on real-world user feedback.

Lastly, the Story Mosaic system was trialed with a limited number of caregivers from a single nursing home. Its efficiency and effectiveness might differ with a broader set of caregivers who possesses varied experiences. Future endeavors will engage a wider and more diverse group of caregivers across multiple nursing facilities. Gleaning insights from this expanded user group will be instrumental in fortifying the system’s algorithms and features, ensuring its reliability and versatility across varied contexts.

### Conclusion

As the global aging population continues to grow, the importance of tools such as the Story Mosaic system becomes increasingly evident. This system, designed to empower caregivers, capitalizes on advanced digital technology, participatory design, and iterative feedback. Consequently, the Story Mosaic system emerges as a trailblazer in weaving personal narratives into contemporary caregiving. The Story Mosaic system streamlines information extraction, automates organization, and improves narrative management for caregivers to better understand and provide personalized care to older adults by organizing life stories intuitively, fostering ongoing enrichment through dynamic interactions. Given the wealth of memories and experiences that older adults contribute, it is crucial to keep their stories at the heart of caregiving innovations. Looking forward, expanding the scope of the Story Mosaic system to encompass informal caregivers and family members holds promise. This expansion ensures that the continued evolution of the Story Mosaic system remains as adaptable and empathetic as the caregivers who will come to depend on it.

## Supplementary material

10.2196/50037 Multimedia Appendix 1 The mapping relationship between user requirements and prototype functions.

10.2196/50037 Multimedia Appendix 2 Usability test plan.

10.2196/50037 Multimedia Appendix 3 Initial design requirements.

10.2196/50037 Multimedia Appendix 4 Feedback analysis of the workshops.

10.2196/50037 Multimedia Appendix 5 Problems encountered by participants during usability testing and our proposed fixes.

10.2196/50037 Multimedia Appendix 6 The home page of the Story Mosaic system.

## Abbreviations

HCD: human-centered design
SUS: System Usability Scale
UI: user interface
