# Supplementary material for: Enhancing Caregiver Empowerment Through the Story Mosaic System: Human-Centered Design Approach for Visualizing Older Adult Life Stories
Source: JMIR Aging. 2023 Nov 8;6:e50037. doi: 10.2196/50037 (PMC10662670; doi:10.2196/50037)
Supplement: Multimedia Appendix 1 [file aging-v6-e50037-s001.docx]

**Multimedia Appendix 1**

The mapping relationship between user requirements and prototype functions.

| Category | Requirement ID | Prototype functions |
| --- | --- | --- |
| **Input life stories** | R001 | The system supports manual entry of key components including chronological references, geographic context, people, and event summaries while combining life stories. These informational fields are marked as optional. When these details are not available, the system has an automatic filling algorithm to build in. |
|  | R002 | During the process of inputting life stories, users are afforded the opportunity to incorporate a diverse array of multi-modal formats, encompassing textual narratives, images, and videos, thus enhancing the depth and richness of the life stories. |
| **Organize life stories** | R003 | Users possess the ability to individually customize themes for older adults’ life stories, enabling them to add, delete and modify these themes within the system. |
|  | R004 | If users manually designate a theme for a life story, the system will categorize the story according to the specified theme. In instances where no manual theme designation occurs, the system will automatically categorize the life story using an embedded algorithm. |
|  | R005 | The life stories would be arranged in chronological order from distant to recent, based on the time element. |
|  | R006 | The system offers a search box within the life story visualization interface, enabling users to engage in a nuanced search of life stories through a process of approximate matching. |
|  | R007 | Calculate the number of life stories under each theme. When displaying topics, the prominence of a theme will be proportional to the number of stories associated with it. |
| **Generate Timeline** | R008 | Display the event summaries of each life story in the visualization interface, presented in the format "Time: Event Summary". |
|  | R009 | Arrange the event summaries of older adults in chronological order to generate a timeline of their life experiences. |
| **Information management** | R010 | The module of the information management is set up in the prototype to support the operation of adding, deleting, correcting, and checking older adults’ information. |
|  | R011 | In addition to the basic population information, care information is set in the elderly information, including health status, dietary contraindications, care precautions and other fields. |
