# Supplementary material for: Enhancing Caregiver Empowerment Through the Story Mosaic System: Human-Centered Design Approach for Visualizing Older Adult Life Stories
Source: JMIR Aging. 2023 Nov 8;6:e50037. doi: 10.2196/50037 (PMC10662670; doi:10.2196/50037)
Supplement: Multimedia Appendix 2 [file aging-v6-e50037-s002.docx]

## Multimedia Appendix 2

Usability test plan

**Tasks**

1. Login: Please enter your account number and password to log in to the Story Mosaic system.

2. Add a new older adult:

Name: Mr. Wang

Gender: Male

Contact Information: 13033011111

ID Card Number: 370***********6211

Address: Hibiscus Community Ringside Subdivision

Hobbies: Enjoys playing chess and practicing calligraphy.

Dietary Preferences: Needs to control sugar intake due to high blood sugar; Prefers a mild and light diet, avoids onions and coriander.

Special Notes: Takes a designated rest period from 1 pm to 2 pm, and during this interval, we kindly ask for consideration to avoid disturbances. Demonstrates a preference for solitude, actively appreciating an atmosphere of tranquility.

3. Add a new topic:

Older adult: Mr. Wang

Topic: Achievement

4. Add a new story:

Older adult: Mr. Wang

Story Details:

Year: 1935

Description: Mr. Wang enrolled in a private educational institution to pursue his academic endeavors. This establishment was conveniently situated near his residence, and fortuitously, the school's headmaster, Mr. Yang, happened to be a familial relation. As per the customary practice, each day upon their arrival, the students would conduct formal bows at designated areas within the school premises, followed by a display of reverence towards the esteemed master. Within the confines of this private scholastic environment, Mr. Wang's daily regimen predominantly centered on scholarly pursuits encompassing reading, diligent study, and the cultivation of calligraphic skills.

5. Timeline Viewing: Locate the older adult "Mr. Wang," and select the "Career "theme. Then please view the timeline for this theme in relation to Mr. Wang.

6. Life Story Viewing: View the details of the life story under the first event node in the Mr. Wang’s timeline.

7. Retrieval: In the life story viewing interface for Mr. Wang, enter "visiting relatives" in the retrieval box, then click the "Search" button to retrieve all content related to "visiting relatives" in Mr. Wang 's life stories.

**Closing Questions**

Q1. Have the life stories of Mr. Wang within the Story Mosaic system provided you with a deeper understanding of him?

Q2. Does the Story Mosaic system relieve your pressure to organize and consult life stories?

Q3. Are you willing to use the Story Mosaic system in your work?
