# Supplementary material for: Enhancing Caregiver Empowerment Through the Story Mosaic System: Human-Centered Design Approach for Visualizing Older Adult Life Stories
Source: JMIR Aging. 2023 Nov 8;6:e50037. doi: 10.2196/50037 (PMC10662670; doi:10.2196/50037)
Supplement: Multimedia Appendix 3 [file aging-v6-e50037-s003.docx]

## Multimedia Appendix 3

Initial design requirements identified from a follow-up observation and five semi-structured.

| Category | Requirement ID | Requirement details | Representative quotes |
| --- | --- | --- | --- |
| **Input life stories** | R001 | This system needs to support the highlighting of important elements of life stories such as time and location. | A1: Time is very important information, such as older adults' birthdays, anniversaries and so on. |
|  | R002 | The system should support the addition of multimodal life stories such as text, pictures, and videos. | C2: Although most of the life stories are narrated by older adults, sometimes they provide photographs that correspond to the life stories. |
| **Organize life stories** | R003 | This system should support the presupposition of life story themes, such as career, achievements, family, and more. | C1: Older adults have different tendencies when telling their life stories. Some older adults with high achievements like to tell their career achievements, such as "making cannonballs"; Older women were more likely to talk about their children. |
|  | R004 | The system needs to support the automatic categorization and organization of older people's life stories by themes. | A1: We usually simply record the life story of the elderly and then save it in one document or multiple documents. We don't have time to sort them out. |
|  | R005 | The system needs to be able to automatically sort life stories in chronological order. | C3: It takes a special amount of time to organize these life stories. We spend most day caring for older adults and do not have much time to organize them. For now, we can only record the life stories and then ask volunteers to organize the life stories chronologically when they come to volunteer activities. |
|  | R006 | The system should facilitate the retrieval of life stories. | C2: With the deepening of communication, there are more and more life stories of the elderly, and it is difficult for us to find a special life story quickly |
|  | R007 | The system needs to be able to learn the life stories of older adults and visualize the information that is useful for care. | C3: Older adults usually talk about whatever comes to their mind. It's hard enough to keep track of the stories they tell. As more and more life stories are told, it becomes more and more difficult for us to organize and revisit them. |
| **Generate Timeline** | R008 | This system needs to support simplifying redundant life stories and provide the crucial events of each life story. | C1: The life stories narrated by older adults are rich in content, which makes it difficult for us to quickly find the crucial information. |
|  | R009 | The system needs to provide a view to visualize the main life experience of the older adult. | A2: We want to build a digital health profile of an older adult based on their life story, much like a work experience profile on a resume. |
| **Information management** | R010 | The system needs to support the modification of the older adult. | C2: Information about older adults sometimes changes, such as their medical history, care precautions, and so on. |
|  | R011 | The system needs to support older adults’ interests, health, diet, and other care information. | A2: Gaining insights into dietary considerations and medical history of older adults enhances our caregiving towards them. |
