# Supplementary material for: Enhancing Caregiver Empowerment Through the Story Mosaic System: Human-Centered Design Approach for Visualizing Older Adult Life Stories
Source: JMIR Aging. 2023 Nov 8;6:e50037. doi: 10.2196/50037 (PMC10662670; doi:10.2196/50037)
Supplement: Multimedia Appendix 4 [file aging-v6-e50037-s004.docx]

## Multimedia Appendix 4

Feedback Analysis of Workshops.

| Category | Suggestions | Representative quotes |
| --- | --- | --- |
| **Feedback on user interface and usability** | Enhancing the navigational transition from subsequent pages back to the main "home" page should embody a heightened level of intuitiveness, aligning with the principle of "User control and freedom." | C2: What actions should be taken to swiftly return from the interface displaying the life story of an older adult and proceed to explore the life story of another one? |
|  | Operation buttons need to be obvious and use a combination of text and icons, aligning with the principle of “Flexibility and efficiency of use”. | C2: Does this magnifying glass icon mean a search button? |
|  | The consolidation of personal information and life narratives pertaining to older adults should be manifested within a unified interface, aimed at alleviating the cognitive burden imposed on the user's memory retrieval processes, aligning with the principle of “Recognition rather than recall”. | C4: From which older adult is the life stories? Would it be possible to provide additional information about him on the same interface, sparing me the need to navigate through multiple interfaces to obtain the information? |
|  | Enlarged font for older caregivers, aligning with the principle of “Aesthetic and minimalist design”. | C1: I am experiencing some difficulty in reading the densely packed text due to my presbyopia eyesight. |
|  | Informative cues necessitating distinct color depictions, aligning with the principle of “Help and documentation”. | D1: You want to highlight the different meanings of different life stories. Here I suggest you consider using a different color background. |
| **Optimization suggestions for existing features** | Enhance the discernibility of hierarchical information within the life story presentation. Currently, the hierarchy encompassing themes, events, and story particulars lacks clarity and appears somewhat congested. | D1: Can you try to add a timeline icon in front of the time point of the event, so that it is better to see the chronological order. |
|  | Integrate the life stories and timeline of older adults into a unified module, thereby minimizing the need for distinct user interactions. This approach aims to seamlessly amalgamate these aspects and streamline user engagement. | C4: The life story and timeline could be consolidated within a single module. When nodes are collapsed, the timeline would be visible, and upon expansion, detailed life story information would become accessible. |
|  | Themes can be visually highlighted using varying colors and font sizes, reflective of their content depth. However, the current narrative organization structure within the story display interface may not effectively convey the emphasis of older adults' experiences. | C5: Highlight different topics with colors and bigger letters, but make sure it's easy to understand. |
|  | Enhance the foundational information fields for elderly individuals. Consider incorporating elements such as religious affiliations, household registration, dietary constraints, and health status into the fundamental senior profile. This augmentation would contribute significantly to tailoring their care regimen. | C5: I believe that aspects such as the older adult's religious beliefs and medical history are also of significant importance. For instance, if an older adult adheres to the Christian faith, it could be beneficial to arrange a weekly place of worship for them. |
|  | Integrate the Life Story and Elder Modules within the menu interface. Upon selecting the profile of elderly individuals, the option to explore their visual life narrative should seamlessly emerge. | C4: Put Life Story and Elder Modules in one place in the menu. When we click on a person, their story should come up. |
| **Request for additional functionalities** | Incorporate a "Story Management" function to the system, facilitating the adding and editing of life stories. | D2: I think we need a special storage module for life stories, like a repository. These stories might not have to show up on the elderly person's visual interface right away, but we should have a place to store and edit them. |
|  | Memoir making. Facilitate the creation of electronic memoirs through the selection of life stories, allowing users to curate personalized narratives of older adults, accessible for viewing by both seniors themselves and their family members. | D2: Can these life stories be exported and turned into an electronic memoir for the elderly? |
|  | The system should provide the capability to establish secondary accounts. | C1: Can we create accounts for the older adults' children too? This could let them see our efforts for their parents and help strengthen parent-child bonds through exploring life stories. |
