# Supplementary material for: Enhancing Caregiver Empowerment Through the Story Mosaic System: Human-Centered Design Approach for Visualizing Older Adult Life Stories
Source: JMIR Aging. 2023 Nov 8;6:e50037. doi: 10.2196/50037 (PMC10662670; doi:10.2196/50037)
Supplement: Multimedia Appendix 5 [file aging-v6-e50037-s005.docx]

## Multimedia Appendix 5

Problems encountered by participants during usability testing and our proposed fixes.

| Category of Problem | Issue(s) | Fix(es) |
| --- | --- | --- |
| **Interaction** | The user couldn’t recognize the magnifying glass search button. | Use a more intuitive icon for the search button, such as a magnifying glass icon accompanied by the word "Search" or a distinctive color. Provide a hover effect or animation to make it stand out. |
|  | Did not click on the view button but clicked on the story text. | Increase the click area of the "View" button or make it visually distinct. Add a subtle animation or highlight to draw attention to the button. |
|  | Older people with the same name, need to enter the details screen to distinguish. | Include additional identifying information, such as profile pictures or birthdates, on the details screen to help users differentiate between individuals with the same name. |
|  | Difficult to find the target event. | Implement a robust search and filtering system. Allow users to filter events by date, location, keywords, and other relevant criteria. Provide clear labels and tooltips to guide users in using the search and filter features. |
| **Bootstrap operation**  **Input errors** | Not found the current interface directly back to the home page navigation. | Include a consistent navigation element (e.g., a logo or "Home" button) that users can easily identify and click to return to the home page from any interface or section. |
|  | Slide to the bottom of the page, ignore the direct return to the top of the page button. | Implement a floating "Return to Top" button that remains visible as users scroll down the page. Use a distinct color or icon to make it easily noticeable. |
|  | Not all requested fields were entered. | Implement real-time form validation that highlights missing fields and provides descriptive error messages. Clearly indicate required fields with asterisks or other visual cues. |
| **Complex operation** | When uploading stories in batches, the format is wrong, resulting in upload failure. | Provide clear guidelines for the required story format before users attempt batch uploads. Offer a sample file and validate the format before uploading. Display informative error messages for format-related upload failures. |
|  | Incorrect password input multiple times. | Remove the requirement of the simultaneous presence of case and symbols in the password. Provide clear instructions on password recovery and reset options. |
