# Supplementary figures and images for: Enhancing Caregiver Empowerment Through the Story Mosaic System: Human-Centered Design Approach for Visualizing Older Adult Life Stories
Source: JMIR Aging. 2023 Nov 8;6:e50037. doi: 10.2196/50037 (PMC10662670; doi:10.2196/50037)

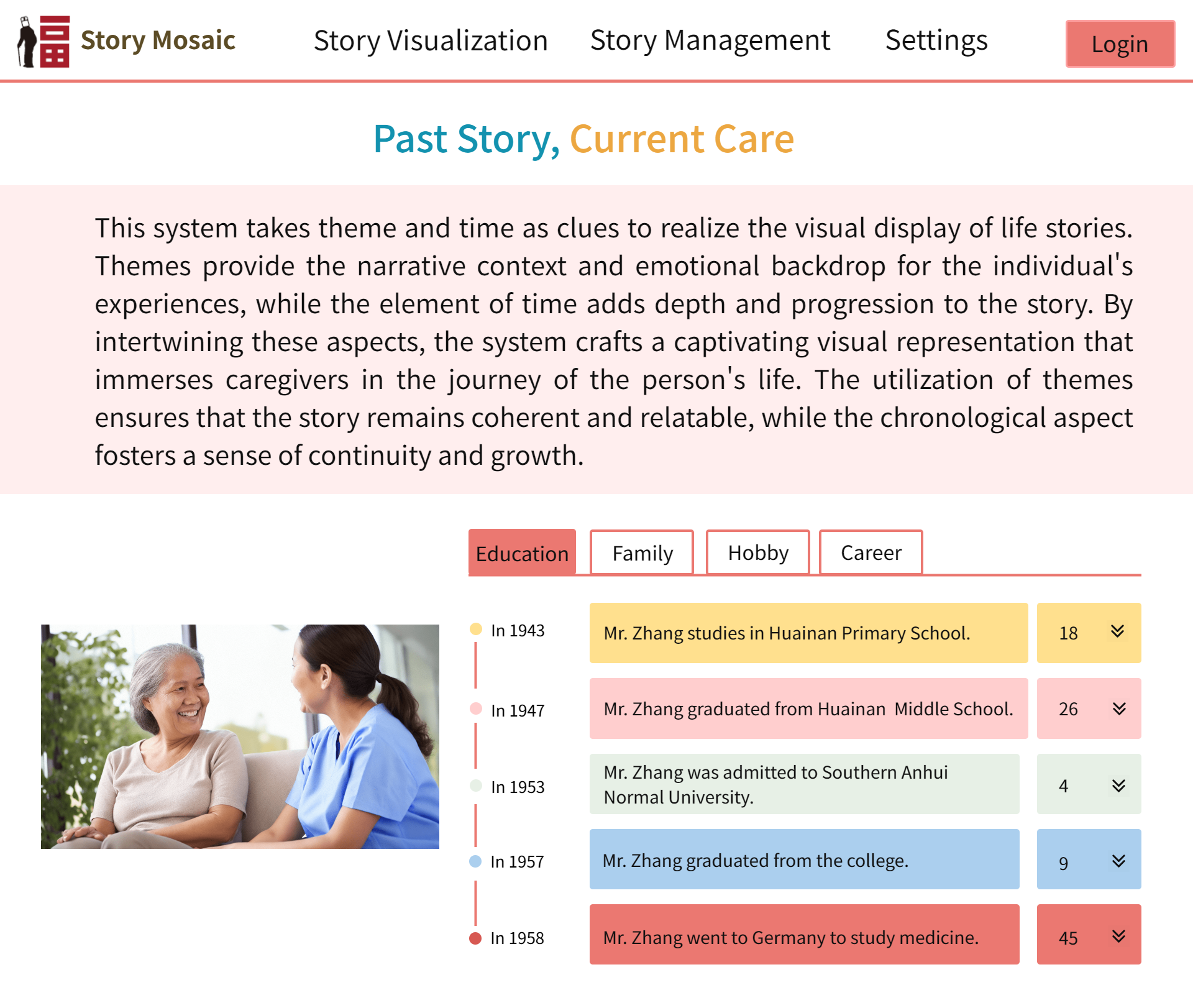

Supplement: Multimedia Appendix 6 [file aging-v6-e50037-s006.png]
